# Allelopathy of Bracken Fern (*Pteridium arachnoideum*): New Evidence from Green Fronds, Litter, and Soil

**DOI:** 10.1371/journal.pone.0161670

**Published:** 2016-08-23

**Authors:** Luciana de Jesus Jatoba, Rosa Maria Varela, José Maria Gonzalez Molinillo, Zia Ud Din, Sonia Cristina Juliano Gualtieri, Edson Rodrigues-Filho, Francisco Antonio Macías

**Affiliations:** 1 Laboratory of Plant Phytochemical and Reproductive Ecophysiology Studies, Department of Botany, Universidade Federal de São Carlos (UFSCar), São Carlos, São Paulo, Brazil; 2 Cádiz Allelopathy Group (GAC), Organic Chemistry Department, Facultad de Ciencias, Universidad de Cádiz (UCA), Puerto Real, Cádiz, Spain; 3 Laboratory of Micromolecular Biochemistry of Microorganisms (LaBioMMi), Chemistry Department, Universidade Federal de São Carlos (UFSCar), São Carlos, São Paulo, Brazil; Northwest Agriculture and Forestry University, CHINA

## Abstract

The neotropical bracken fern *Pteridium arachnoideum* (Kaulf.) Maxon. (Dennstaedtiaceae) is described as an aggressive pioneer plant species. It invades abandoned or newly burned areas and represents a management challenge at these invaded sites. Native to the Atlantic Forest and Cerrado (Tropical Savanna) Brazilian biomes, *P*. *arachnoideum* has nevertheless become very problematic in these conservation hotspots. Despite some reports suggesting a possible role of allelopathy in this plant’s dominance, until now there has been little evidence of isolated and individually identified compounds with phytotoxic activities present in its tissues or in the surrounding environment. Thus, the aim of this study was to investigate the allelopathic potential of *P*. *arachnoideum* by isolating and identifying any secondary metabolites with phytotoxic activity in its tissues, litter, and soil. Bioguided phytochemical investigation led to the isolation and identification of the proanthocyanidin selligueain A as the major secondary compound in the green fronds and litter of this fern. It is produced by *P*. *arachnoideum* in its green fronds, remains unaltered during the senescence process, and is the major secondary compound present in litter. Selligueain A showed phytotoxic activity against the selected target species sesame (*Sesamum indicum*) early development. In particular, the compound inhibited root and stem growth, and root metaxylem cell size but did not affect chlorophyll content. This compound can be considered as an allelochemical because it is present in the soil under *P*. *arachnoideum* patches as one of the major compounds in the soil solution. This is the first report of the presence of selligueain A in any member of the Dennstaedtiaceae family and the first time an isolated and identified allelochemical produced by members of the *Pteridium* species complex has been described. This evidence of selligueain A as a putative allelochemical of *P*. *arachnoideum* reinforces the role of allelopathy in the dominance processes of this plant in the areas where it occurs.

## Introduction

*Pteridium* plants are cosmopolitan heliophyte ferns that are among the most abundant plant taxa on the planet, present on all continents except Antarctica [1,2]. Plants of the *Pteridium* genus exhibit extensive phenotypic plasticity with many morphological intermediates between definable morphotypes and the ability for these to hybridize over long distances. Until 2009, the genus was considered monotypic with two subspecies (*Pteridium aquilinum* ssp. a*quilinum* and *Pteridium aquilinum* ssp. c*audatum*). Eight varieties were recognized within subspecies *aquilinum*, all found in Europe, and four were recognized within subspecies *caudatum*, found in South and Central America, Asia, and Oceania [1,2]. A growing need for the revision of the systematics of this species complex culminated in the proposal and adoption of a new infrageneric taxonomic classification based on an analysis of chromosome ploidy and mitochondrial DNA. *Pteridium arachnoideum* is a separate species according to this classification [1]. In this study, the most recent nomenclature has been adopted, although many of the references on the biology, ecology, and phytochemistry of *Pteridium* species adhere to the previous classification system.

*Pteridium* ferns represent a challenge to biodiversity management and conservation around the world as they become dominant in degraded or anthropogenic impacted areas [3,4] and change the environment significantly. They affect soil physicochemical parameters and the cycling and availability of nutrients [5]. The large amount of litter they produce acts as fuel biomass, contributing to large, hot fires [6]. They also represent a challenge in the areas of environmental restoration by interfering with the seed bank composition [7], resprouting of species of interest [6], and vegetation restoration dynamics [8]. The neotropical bracken fern *Pteridium arachnoideum* (Kaulf.) Maxon. (Dennstaedtiaceae) is described as an aggressive pioneer species that is difficult to manage in the abandoned or newly burned areas it invades [2,9]. It has become problematic in the Atlantic Forest and Cerrado (Tropical Savanna) Brazilian biomes, both of which are classified as conservation hotspots [10]. Its effects on these biomes include seed bank depletion [11], interference with tree species establishment in the Atlantic Forest [9], and interference with the vegetation structure of the Tropical Savanna [12].

Understanding the mechanisms that govern plant dominance and the success of invasive plants has been one of the greatest scientific challenges of the recent decades [13]. These mechanisms involve ecological and evolutionary processes [14], including interactions mediated by resources and competition in the strict sense [15] as well as interactions such as allelopathy [16]. Allelopathy is defined as any direct or indirect effect on the growth, survival, or reproduction of a target plant mediated by the secondary metabolites produced by a donor plant and released into the environment [17].

Secondary metabolites with allelopathic potential are known as allelochemicals. Allelochemicals produce environmental modifications and phytotoxic effects directly via phytotoxicity, or indirectly [18] by interfering with soil microbiota [19], nutrient cycling dynamics and availability [20], or other processes. In this scenario, the isolation and identification of chemical compounds with experimentally observed activities is an essential step in confirming allelopathy [21,22].

Many studies on the phytochemistry of *Pteridium* species have been performed because of pharmaceutical and toxicological interest in their secondary metabolites. In particular, there has been research concerning pterosins and pterosides activities, such as carcinogenic activity, and of the sesquiterpene ptaquiloside, which is the compound principally responsible for the carcinogenic effects of *P*. *aquilinum* in mammals [2,23]. These plants produce large amounts of secondary metabolites with diverse, but not yet fully understood, biological activities [2] and become dominant and problematic in the impacted areas where they occur. However, the mechanisms by which they attain this dominance are not yet well established. Allelopathic interactions can play an important role in this phenomenon, but despite reports on the possible role of allelopathy in this plant’s dominance [9,24], no compounds with phytotoxic activity or any activities associated with allelopathic interactions have been isolated or individually identified for *P*. *arachnoideum* or any *Pteridium* species.

Thus, the aim of this study was to investigate the allelopathic potential of *P*. *arachnoideum* by isolating and identifying secondary metabolites with phytotoxic activity in its tissues, and the availability of such metabolites in the surrounding environment.

## Materials and Methods

### Initial Extraction and Bioguided Isolation

#### Plant Material

Plant material comprising green fronds, litter, and rhizomes of *P*. *arachnoideum* was collected at the end of the dry season from the Tropical Savanna reserve area of the Federal University of São Carlos (UFSCar), São Paulo State, Brazil (21°58'02.0" S, 047°52'09.9" W). A voucher specimen was deposited at the herbarium of the UFSCar Botany Department (Voucher n° HUFSCAR8797). Following inspection to remove visible damaged or fungal infected material, it was washed, and green fronds and litter were dried in the shade in a greenhouse (17–43°C). Rhizomes were dried in an oven with forced air circulation at 40°C until a constant weight was reached (190 h). Plant material was ground in an electric mill, and the resulting powder was used for extraction (CITES/FLORA IBAMA Export Permit n° 12BR009485/DF).

#### Equipment and Reagents

Commercially available solvents and reagents were used as supplied. Nuclear magnetic resonance (NMR) was run on 400 MHz, 500 MHz, and 600 MHz (Agilent, USA) or 400 MHz (Bruker, USA) spectrometers. Chemical shifts were given in ppm, referenced to the deuterated methanol solvent (Methanol-*d4*) residual ^1^H signal (*δ* 4.78), or with tetramethylsilane as internal standard. Analysis by thin layer chromatography (TLC) was carried out using Alugram Sil G/UV_254_ eluted with ethyl acetate (EtOAc), and Alugram RP-18 W/UV_254_ plates (both Macherey-Nagel, Germany) eluted with H_2_O/methanol (MeOH) (5:2 v/v) or H_2_O/MeOH/Acetonitrile (ACN) (5:1:1 v/v). Column chromatography was performed using Lichroprep RP-18 (40–63 μm; Merck, Germany), Discovery DSC-18 (50 μm; SUPELCO, Sigma-Aldrich, USA), and silica gel 0.060–0.200 mm, 60 Å (Acros Organics, Belgium). Flash chromatography was performed on a CombiFlash Rf apparatus using a 15.5 g RediSep Rf Gold C18 column (both Teledyne Isco, USA). High pressure liquid chromatography (HPLC) coupled with a refractive index (RI) detector (HPLC-RI; Merck-Hitachi, Japan) was performed using LiChrospher RP-18 (10 μm, 250 × 10 mm; Merck, Germany) and Gemini C18 (5 μm, 250 × 4.60 mm; Phenomenex, USA) columns. HPLC coupled with photodiode array (PDA; HPLC-PDA) detector was performed in Waters Alliance 2695 chromatographic system coupled with a Waters 2995 PDA detector (USA), using a Luna 5-μm Phenyl-Hexyl column (5 μm, 250 × 4.6 mm; Phenomenex, USA).

#### Extraction and Isolation

Each plant material sample was extracted using 100 g of dried powder and 500 mL of hexane (Hex) in an ultrasonic bath for 15 min to remove fats and waxes. Following vacuum filtration, this extraction procedure was repeated four times. Defatted material was subjected to five extractions with EtOAc and five extractions with MeOH [25], yielding two extracts each for green fronds (EtOAc, 1.12 g; MeOH, 30.86 g), litter (EtOAc, 455.8 mg; MeOH, 4.80 g) and rhizomes (EtOAc, 255.3 mg; MeOH, 6.78 g).

The EtOAc extracts were first subjected to reverse phase chromatography for chlorophyll removal using a gradient with increasing amounts of MeOH in water (pure water; H_2_O/MeOH 5:1, 5:2, 5:3, 5:4 v/v; pure MeOH) followed by pure dichloromethane [25]. After TLC analysis, four fractions were obtained from green fronds (GF-1–GF-4) and litter (L-1–L-4).

Fractions GF-2 and L-2 were subjected to normal phase chromatographic processes in a silica gel column eluted using a gradient with increasing amounts of acetone in hexane (Hex/Acetone 1:1, 5:3, 10:7; 5:4; pure acetone) followed by pure MeOH to obtain multiple subfractions: GF-2A–GF-2E and L-2A–L-2D.

Fractions GF-2B and L-2C were subjected to reverse phase HPLC-RI using a semi-preparative column and isocratic elution with ACN/H_2_O (20:7 v/v) to obtain the following subfractions: GF-2B.1 and GF-2B.2 (from GF-2B), and L-2C.1 and L-2C.2 (from L-2C). Subfraction GF-2B.2 was purified by reverse phase HPLC-RI using an analytical column and isocratic elution with ACN/H_2_O (1:5 v/v). Crude extracts and fractions were subjected to ^1^H-NMR analysis, and the purified fraction GF-2B.2p was subjected to additional ^13^C, COSY-^1^H-^1^H, HMBC, and HSQC NMR analyses.

#### Wheat Coleoptile Bioassays

The phytotoxic activities of the fractions were measured by conducting bioassays using fragments of etiolated wheat coleoptiles. Wheat seeds (*Triticum aestivum* L. cv. Catervo) were distributed on Petri dishes (20-mm diameter) lined with a sheet of filter paper moistened with 5 mL of distilled water. Petri dishes were then wrapped in aluminum foil and placed in a germination chamber for 72 h at 25 ± 1°C. Under a safe green light, the apical 2 mm of each etiolated wheat seedling was cut off and discarded. The next 4 mm of each coleoptile was collected for bioassay [25]. Wheat seeds were donated by the Agricultural and Fishery Research and Instruction Institute of Spain (IFAPA Centro Rancho de la Merced, Jerez de la Frontera, CA, Spain). The fractions tested were presolubilized in dimethyl sulfoxide (DMSO) and diluted with a potassium phosphate buffer solution (250-mL distilled water, 5-g sucrose, 0.2625-g citric acid, and 0.725-g dibasic potassium phosphate; pH 5.6) to obtain solutions of 0.8, 0.4 and 0.2 mg.mL^−1^ with a constant DMSO concentration (5 μL.mL^−1^). For the bioassay conducted using isolated selligueain A, solutions of 1, 0.3, 0.1, 0.03, and 0.01 mM were prepared as previously described [25]. An herbicide treatment was adopted using Logran Extra (terbutryn 59.4% + triasulfuron 0.6%; Syngenta Agro, Spain) solubilized in buffer solution at the same concentrations used for fractions or isolated selligueain A and DMSO (5 μL.mL^−1^). Negative control was performed using buffer solution and DMSO (5 μL.mL^-1^). Bioassays were prepared in triplicate for each treatment by adding 2 mL of each solution and five wheat coleoptile fragments to each test tube. Test tubes were capped and kept in the dark in a germination chamber under rotation (0.25 rpm) at 25 ± 1°C [25]. After 24 h, the tubes were opened and the coleoptiles were photographed and measured using Photomed 4 software (Universidad de Cádiz, Puerto Real, Spain).

### Soil Analysis

Samples of surficial soil (15 cm in depth) were collected at the start of the dry season from under a *P*. *arachnoideum* patch in the Tropical Savanna reserve area of UFSCar, São Paulo State, Brazil (21°58'02.0" S, 047°52'09.9" W). Soil samples were placed in food plastic bags and transported immediately to the laboratory for extraction. Four soil samples (ESQ-1–ESQ-4) were collected (0.58 ± 0.18 kg) from locations spaced at least two meters apart.

Prior to extraction, each sample was inspected to remove visible root, leaf, stone, and arthropod fragments. The extraction was performed for each sample with 50 g of fresh soil and 200 mL of MeOH, under constant agitation in an orbital shaker (120 rpm) for 18 h [26]. The extract was then filtered with qualitative filter paper and dried under constant air flow. The dried extract was suspended in 4 mL of MeOH and filtered with a Minisart^®^ syringe filter (hydrophilic, cellulose acetate, 28 mm, 0.2-μm pore diameter, Sartorius Stedim Biotech, Germany) to remove fine particles and dried under constant air flow.

Soil extracts were then solubilized in 4 mL MeOH and analyzed by TLC (eluted with H_2_O/MeOH 5:2 v/v) and HPLC-PDA for detection of selligueain A. HPLC-PDA was performed using gradient elution with increasing amounts of ACN (solvent B) in water (solvent A). The gradient profile was as follows: 0–20 min, linear 20–30% of B; 20–25 min, linear 30–100% of B; 25–30 min, linear 100–20% of B. Soil analysis was performed by separate injections of the isolated selligueain A standard and soil extracts in succession.

The selligueain A used as a standard for the soil extract analyses and early growth bioassays was obtained from the EtOAc litter extract of *P*. *arachnoideum* by flash chromatography as follows. Litter material was extracted as previously described, and after the first reverse phase chromatographic separation, the corresponding sample L-2 was subjected to reverse phase flash chromatography with 214-nm ultraviolet (UV) light monitoring using gradient elution with increasing amounts of MeOH (solvent B) in water (solvent A) to provide the desired compound. The gradient profile was as follows: 0–10 min, linear 0–10% of B; 10–20 min, linear 10–40% of B; 20–30 min, linear 40–70% of B; 30–45 min, linear 70–100% of B. Selligueain A was collected as a peak at 22 min (40% of B; H_2_O/MeOH 5:2 v/v).

### Early Growth Bioassay

The phytotoxicity of selligueain A was tested on sesame seedlings (*Sesamum indicum* L. var. BRS seda) early growth as a measure of its allelopathic potential. Phytotoxicity was assessed by measuring morphological and biochemical parameters that can influence competitiveness in field conditions, such as stem and root length and chlorophyll content [27,28]. As this bioassay was intended to serve as the first assessment of selligueain A phytotoxicity to plant early development, sesame was chosen as the target species because of its genetic homogeneity, which tends to result in less variance in early development parameters compared with that of the wild species [29]. Sesame seeds were provided by Dr. Nair H. C. Arriel, Brazilian Enterprise for Agriculture Research (EMBRAPA, Campina Grande, PB, Brazil). Selligueain A was solubilized in a 10 mM 2-(N-morpholino)ethanesulfonic acid buffer solution with DMSO (5 μL.mL^−1^) to obtain solutions of 1, 0.3, 0.1, 0.03, and 0.01 mM. The buffer pH was adjusted to 6.00 using 1 M sodium hydroxide (NaOH) solution. A control was performed using buffer solution and DMSO (5 μL.mL^−1^) only [27].

In a germination chamber, sesame seeds were pregerminated in distilled water at 25°C under a 12 h photoperiod. The bioassay was performed with four replicates each of 10 pregerminated seeds (root 2 mm in length) in transparent plastic boxes (11 × 6 × 4 cm) lined with two sheets of qualitative filter paper, moistened with 6 mL of each solution. The boxes were sealed in plastic bags and kept under the pregermination conditions for 7 days [27]. After this time, the stems and roots of the seedlings were measured, photosynthetic cotyledons were removed and stored frozen (-20°C) until chlorophyll extraction, and roots were removed and fixed in 70% ethanol (EtOH) for metaxylem cell evaluation.

#### Chlorophyll Content

Samples of photosynthetic cotyledons (30 mg of fresh weight) were collected and stored frozen in the dark until chlorophyll extraction was performed. The samples collected were one per replicate for each treatment in the early growth bioassay.

Total chlorophyll extraction was performed with the photosynthetic cotyledon samples in sealed test tubes containing 5 mL of DMSO. The test tubes were immersed in water bath at 70°C for 2 h under a safe green light until the photosynthetic cotyledons became visually translucent [30]. Subsequently, the test tubes were kept at room temperature and protected from light until they were cool.

The absorbance of the chlorophyll extracts was measured at 665 nm and 648 nm in a Hach DR 5000 spectrophotometer (Loveland, CO, USA). Results are expressed as chlorophyll a, chlorophyll b, and total chlorophyll in milligrams per gram of fresh tissue, using the following equations [31]:

$$C_{a}=14.85(A^{6}{}^{65})-5.14(A^{6}{}^{48}),$$ (1)

$$C_{b}=25.48(A^{6}{}^{48})-7.36(A^{6}{}^{65}),$$ (2)

$$C_{\left(a+b\right)}=7.94(A^{6}{}^{65})+20.34(A^{6}{}^{48}),$$ (3)

where *C*_*a*_, *C*_*b*_, and *C*_*a* + *b*_ are the chlorophyll a, chlorophyll b, and total chlorophyll concentrations (mg.g^−1^ of fresh tissue), respectively; and *A*^665^ and *A*^648^ are the absorbances at 665 nm and 648 nm, respectively.

#### Metaxylem Cell Evaluation

To assess metaxylem cell growth, the sesame roots of each treatment and of the control of the early growth bioassay were removed and immediately immersed in 70% EtOH. Staining was performed according to a modified Fuchs method [27]. After 7 days in 70% EtOH, the roots were clarified in a 10% NaOH solution at 50°C for 48 h. Roots were then immersed in a 0.25% lacmoid (C_48_H_31_N_3_O_13_) ethanolic solution for 24 h.

Following the staining processes, roots were mounted on glass slides with Apathy’s syrup [27] and left at room temperature in the dark until they were dry. The central region of each root was photographed using an optical microscope (Olympus-BX41) coupled to a digital camera (Sony CCD-IRIS) at 20× magnification. Ten metaxylem cells from each of the four root replicates were measured for each treatment and the control using Image J 1.48v software [32].

### Statistical Analysis

Data from the wheat coleoptile bioassays were evaluated in terms of percentage inhibition or stimulation compared with that of the negative control [25], according to eq (4):

$${\%}_{inhibition/stimulation}=\;\left[\frac{\left(C_{r}\;-\;\bar{C_{t}}\right)\;-\;\left(C_{r}\;-\;\bar{C_{c}}\right)}{C_{r}\;-\;\bar{C_{c}}}\right]\times 100,$$ (4)

where *C*_*r*_ is the length of the reference wheat coleoptile (4 mm); $\overset{-}{C_{t}}$ is the average length of the wheat coleoptile from each treatment; and $\overset{-}{C_{c}}$ is the average length of the wheat coleoptile from the negative control.

The mean inhibitory concentration value (IC_50_) was calculated from the results of the bioassays with the isolated selligueain A, which exhibited over 50% inhibition compared with that of the negative control. This involved adjusting the phytotoxicity data using a logarithmic scale to generate a sigmoidal dose–response curve defined by eq (5):

$$Y=\;Y_{min}\;+\;\frac{Y_{max}\;-\;Ymin}{1\;+\;10^{\left[\left(\text{log}IC_{50}-\;X\right)\;\times \;h\right]}},$$ (5)

where *X* is the concentration logarithm; *Y* is the response (phytotoxicity); *Y*_*max*_ and *Y*_*min*_ are the maximum and minimum values of the response, respectively; and *IC*_*50*_ is the *X* value for the curve that is midway between *Y*_*max*_ and *Y*_*min*_ [27]. The goodness of fit of the data to the three-parameter logistic model (3PL) from the IC_50_ calculation is described by the determination coefficient (R^2^). The curve fitting and IC_50_ and R^2^ value calculations were performed using GraphPad Prism 4.0 software (La Jolla, CA, USA).

Data from the bioassays were subjected to Shapiro–Wilk normality test and Levene’s variance homoscedasticity test. When both requirements were met, the data were analyzed by analysis of variance (ANOVA) followed by Tukey’s mean comparison test. When one or both requirements were not met, the data were analyzed with Kruskal–Wallis nonparametric test, followed by Welch’s t-test mean comparison test.

## Results and Discussion

### Bioguided Isolation

**Fig 1:**
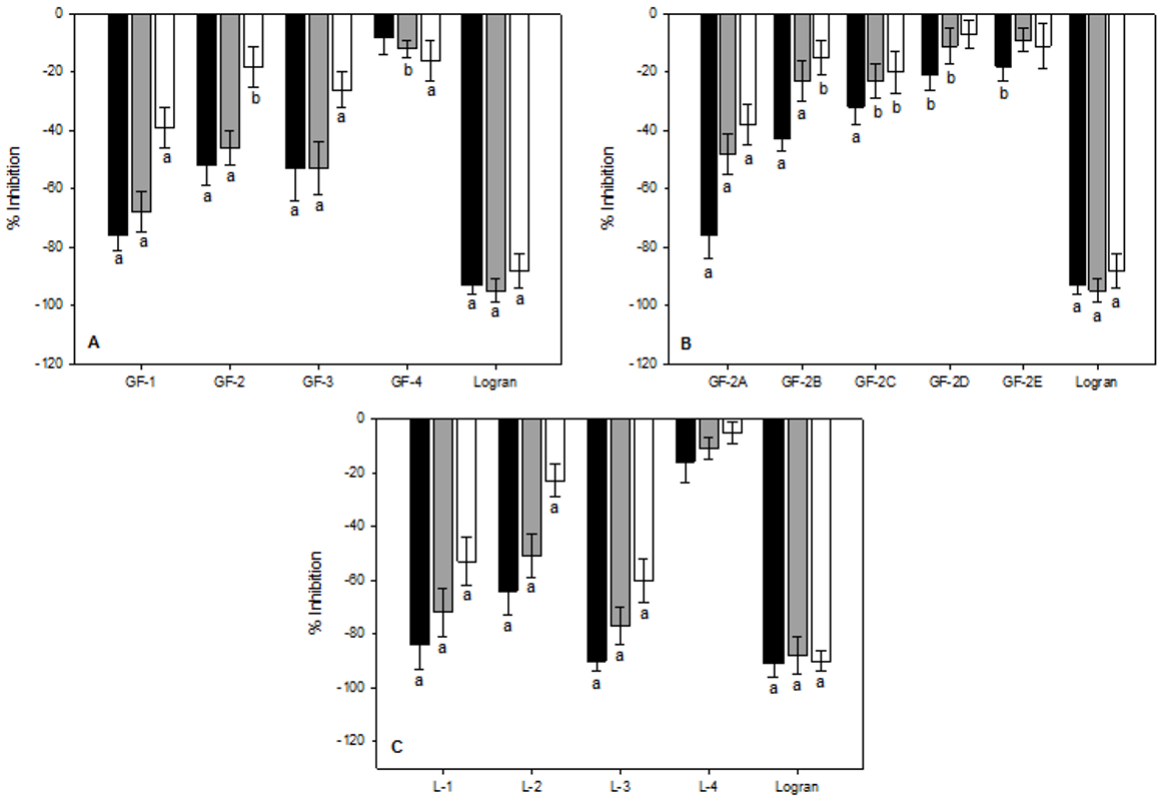
Inhibition of wheat (*T*. *aestivum*) coleoptile fragment elongation in the presence of the EtOAc extracts fractions of *Pteridium arachnoideum* green fronds and litter and the herbicide Logran in different concentrations. (A) and (B): Green frond fractions; (C): Litter fractions. Black columns, 0.8 mg.mL^−1^; gray columns, 0.4 mg.mL^−1^; white columns, 0.2 mg.mL^−1^. Vertical bars indicate standard deviation. Values marked with the letter a (*p* < 0.01) or b (0.01 < *p* < 0.05) are significantly different from those of the negative control according to Welch's test.

For the green fronds extract, fractions GF-1–GF-3 significantly inhibited wheat coleoptile elongation (Fig 1A). Fraction GF-1 (9.26 mg) showed the highest inhibitory activity ranging from −76% to −39% from highest to lowest concentration. The GF-2 and GF-3 fractions (253.3 mg and 56.4 mg, respectively) were also inhibitory, ranging from −52% to −18% for the GF-2 fraction and from −53% to −26% for GF-3 fraction. According to TLC and ^1^H-NMR analysis, these samples contained the same major compound. The compound was the purest in the GF-1 fraction, with an increasing presence of interfering compounds in the subsequent fractions. Given the small yield of the GF-1 fraction, the phytochemical study and bioguided isolation were continued with the GF-2 fraction, which was of greater mass and purer than the GF-3 fraction.

Five subfractions were obtained from the GF-2 fraction (GF-2A–GF-2E) following normal phase chromatography. Of these, the most active fraction was GF-2A (17.3 mg), with inhibitory activity on wheat coleoptile elongation ranging from −76% to −38%, from highest to lowest concentration; followed by GF-2B (69.7 mg), with inhibitory activity ranging from −43% to −15% (Fig 1B). According to ^1^H-NMR analysis, the GF-2A and GF-2B fractions contained the same major compound present in a purer form in the GF-2A fraction. Given the small quantity of GF-2A sample, isolation of the major compound by HPLC-IR was conducted on the GF-2B sample yielding the fraction GF-2B.2p (11.3 mg), a purified compound from green fronds.

**Fig 2:**
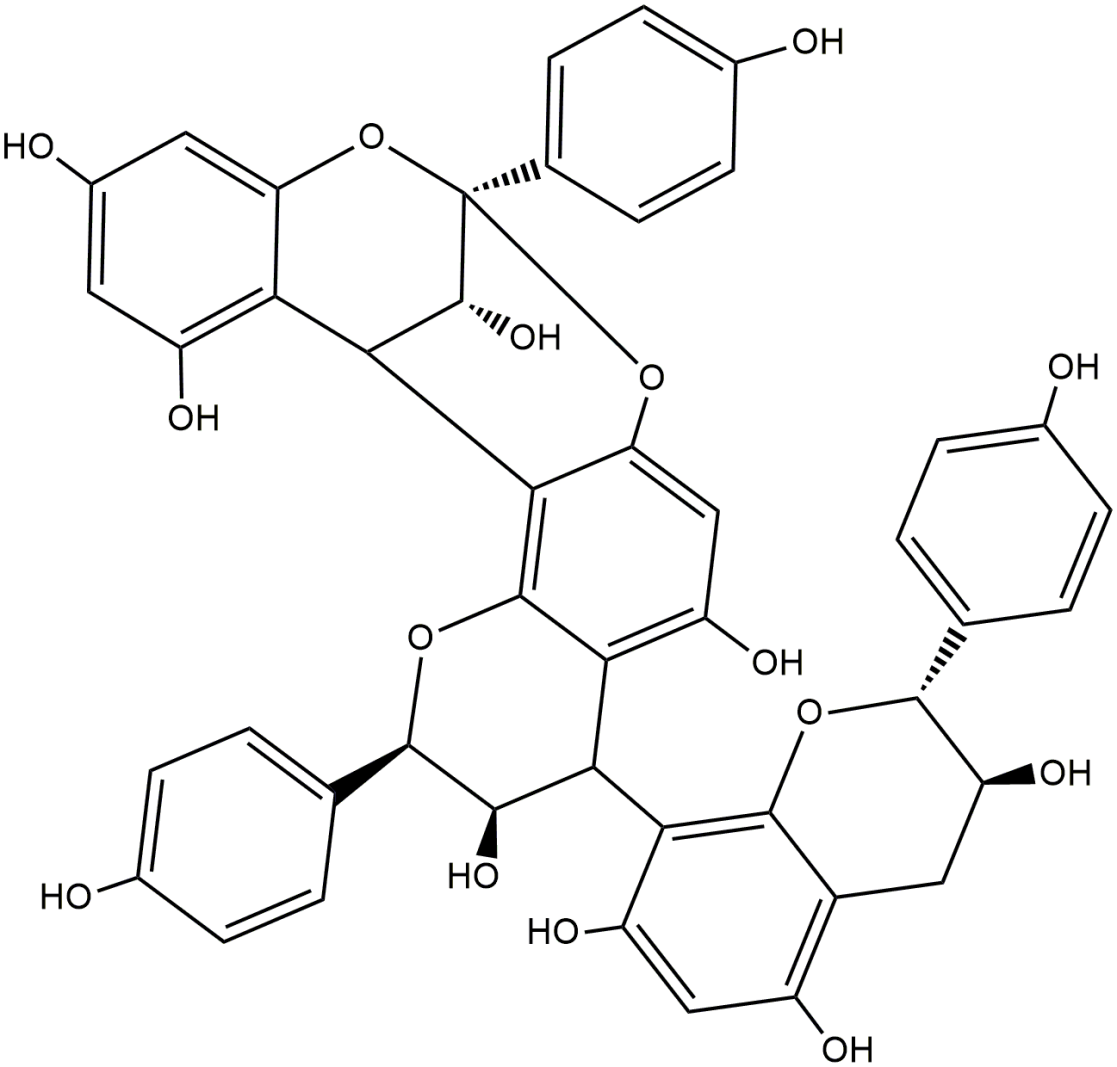
Selligueain A isolated from green fronds and litter of *P*. *arachnoideum*.

An analysis of NMR data combined with spectral results reported in the literature led to the identification of the compound in the GF-2B.2p fraction as proanthocyanidin selligueain A [33,34] (S1 File, Fig 2).

Following the isolation and identification of selligueain A from the green frond extract, a phytochemical study of the litter extract was performed to determine whether the major polyphenolic compound in the litter corresponded to that found in the green fronds or to its derivatives, resulting from the metabolic processes of leaf senescence.

Four fractions were obtained from the EtOAc extract of the litter following reversed phase chromatography (L-1–L-4). Fractions L-1 (9.9 mg) and L-2 (82.1 mg) contained the major compound observed in the crude EtOAc extract, with an increasing presence of interfering compounds in subsequent fractions. The inhibitory activity of fraction L-1 ranged from −84% to −53%, whereas that of fraction L-2 ranged from −64% to −23% (Fig 1C). Fraction L-3 (13.9 mg) also showed strong inhibitory activity ranging from −90% to −60%. This activity was comparable to that of the herbicide treatment, though the major compound of the L-1 and L-2 fractions was not present (Fig 1C). Instead, ^1^H-NMR analysis revealed the presence of flavonol glycosides as major compounds in the L-3 fraction (S2 File).

Given the small yield of the L-1 fraction, the isolation of the major polyphenol compound was performed on the L-2 fraction. Fraction L-2C.1 (6.1 mg) was isolated following normal phase column chromatography and HPLC-RI, and its major compound was identified as selligueain A by NMR analysis (S3 File); the same major compound present in the EtOAc extract of green fronds.

### Proanthocyanidin Selligueain A

Proanthocyanidins are condensed tannins, oligopolymers of three-ring flavonoids with C–C bonds, characterized by the monomers catechin and epicatechin and two hydroxyl radicals attached to ring B [35]. Tannins are considered to be the fourth most important group of biochemical compounds produced by vascular plants, representing a significant portion of the carbon terrestrial biomass [36]. As tannins are complex and costly molecules from an energetic point of view, they are believed to play an important role in plant function and evolution, with activity toward protection against UV radiation [36,37]. Several biological activities have been ascribed to proanthocyanidins and materials rich in these compounds, including antioxidant, anti-inflammatory, and antimicrobial activities [24,38,39]. From an ecological standpoint, condensed tannins are described as having defensive action against herbivory [40] and influencing litter decomposition [41,42], nutrient cycling, and soil microbial activity [43], among other processes.

Selligueain A was first described in the rhizomes of *Selliguea feei* Bory (Polypodiaceae) [33] and is also present in *Polypodium feei* (Bory) Mett., another species of the Polypodiaceae family [44]. This is the first report of selligueain A in *Pteridium arachnoideum* or any member of the Dennstaedtiaceae family, occurring in its green fronds but not its rhizomes.

### Phytotoxic Activity of Selligueain A

Most of the tannins produced by *Pteridium* plants are of the condensed type, proanthocyanidin or prodelphinidin derivatives. Although these tannins are not considered to be among the main toxic compounds produced by these plants, antiherbivore activity has been observed [2,45].

A correlation between the proanthocyanidin concentration and the altitude at which the *Pteridium* plants grow in the Andes region has been observed [37]. This correlation could be an adaptive response to the increased intensity of UV-B radiation at higher altitudes. Analgesic and anti-inflammatory activities have also been attributed to selligueain A following its isolation from *Polypodium feei* rhizomes [44]. However, until now no activities of ecological importance have been attributed to this compound. Selligueain A is present both in *P*. *arachnoideum* green fronds and litter, remaining unaltered during the senescence process. As a resource and an energetically costly compound, its persistence in the litter material indicates putative ecological importance. Therefore, we tested the phytotoxic activity and allelopathic potential of selligueain A using measures of wheat coleoptile elongation and sesame early growth.

**Fig 3:**
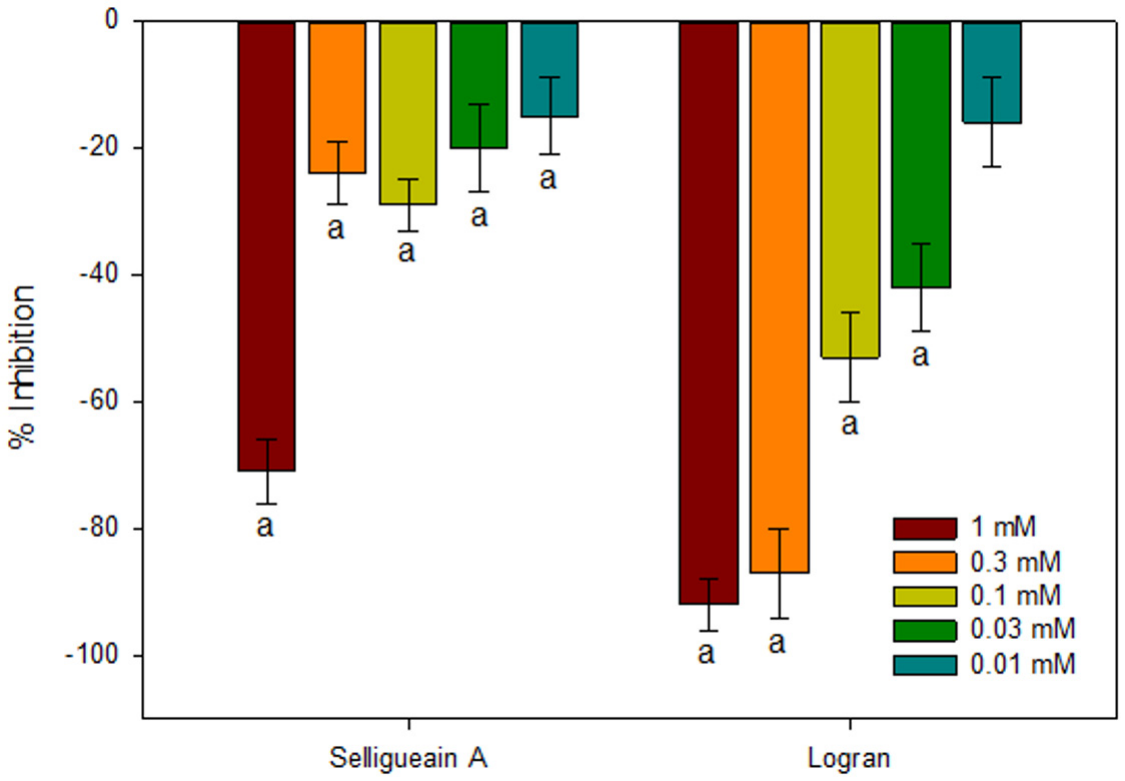
Inhibition of wheat (*T*. *aestivum*) coleoptile fragment elongation in the presence of selligueain A isolated from *P*. *arachnoideum* or the herbicide Logran at different concentrations. Vertical bars show standard deviation. Values marked with the letter a (*p* < 0.01) and b (0.01 < *p* < 0.05) are significantly different from those of the negative control according to Welch's test.

Selligueain A exhibited phytotoxic activity at all concentrations tested, although a significant reduction in activity was observed from the highest to lowest concentrations (Fig 3). At the highest concentration, the inhibitory activity on wheat coleoptile elongation was over −71%, with an IC_50_ value of 0.69 mM (R^2^ = 0.94).

This phytotoxic activity indicates that in addition to protection against UV radiation, proanthocyanidins and other condensed tannins produced by *P*. *arachnoideum* may also possess allelopathic activity.

### Phytotoxicity to Sesame Early Growth

Selligueain A inhibited stem and root development at all concentrations tested. This inhibition ranged from −52% to −34% for stem development and from −46% to −35% for root development (Table 1). Although stem development was inhibited slightly more than root development, potentially affecting the ability to compete for space and light in field conditions, root development inhibition can disrupt water and nutrient intake, which are critical to seedling survival and establishment [46,47].

**Table 1:** Early growth inhibition^a^ of sesame (*S*. *indicum*) seedlings treated with different concentrations of selligueain A.

Selligueain A (mM)	Parameter		
	Shoot Length	Root Length	Metaxylem Cells
% Inhibition			
1.0	-52.22 ± 4.92*	-46.17 ± 1.65*	-50.82 ± 5.49*
0.3	-46.15 ± 1.89*	-43.93 ± 2.96*	-37.88 ± 3.23*
0.1	-44.28 ± 6.35*	-39.38 ± 3.04*	-19.84 ± 4.55
0.03	-34.41 ± 6.14*	-35.94 ± 5.27*	-21.66 ± 4.32
0.01	-38.20 ± 2.29*	-34.69 ± 1.70*	-14.42 ± 5.02
Statistics^b^			
F (p)	41.38 (6.26 x 10^−9^)	58.57 (4.08 x 10^−10^)	13.12 (1.78 x 10^−5^)

Values marked with

* are significantly different from those of the negative control according to Tukey's test (0.01 < p < 0.05).

^a^ % inhibition ± standard deviation

^b^ F = ANOVA F-statistic; p = probability.

Root growth occurs by two main mechanisms: cell division and cell elongation. New cells are produced by rapid cell division in the apical meristem and elongated in the elongation zone of the root tip. In the elongation zone the cells expand, increasing the vacuolar volume and surface area along the root longitudinal axis. This process is under hormonal control and is critical to the development of a functional root system [46]. Here, we chose to evaluate the metaxylem cells because as an anatomic character, they are readily measurable, larger than other root cells, and have thicker cell walls [27], which allows the anatomical evaluation of root cell elongation.

**Fig 4:**
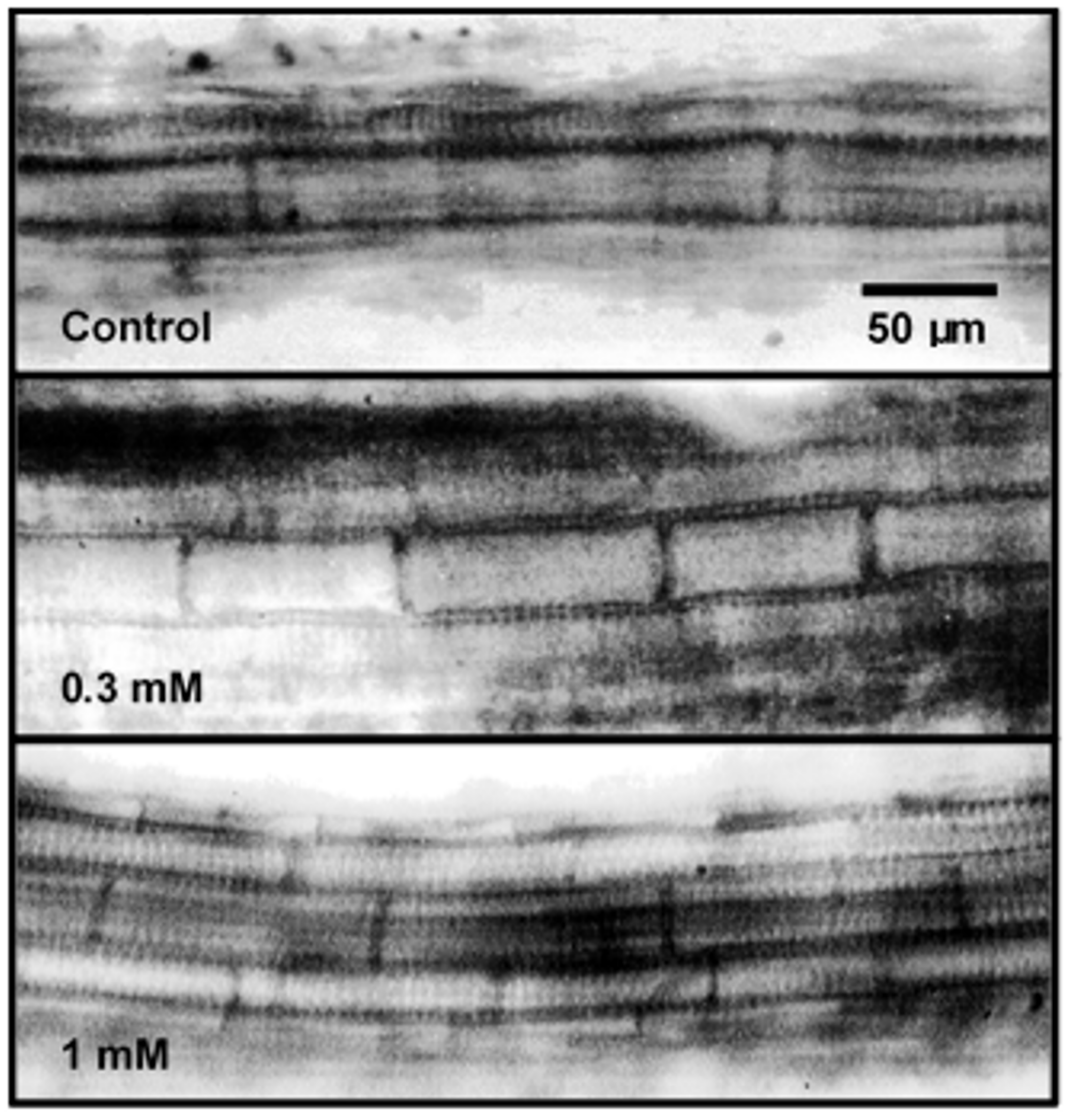
Micrographies of root metaxylem cells of sesame (*S*. *indicum*) seedlings. 20× magnification.

From the size class distribution of the metaxylem cell data (Table 2), a uniform distribution of control cell size was observed, with a greater distribution frequency (40%) of cells between 140 μm and 170 μm. Root metaxylem cells of sesame seedlings grown in the presence of selligueain A were smaller compared with those of the controls. The average metaxylem cell size of the control seedlings was 167 μm. Metaxylem cells of sesame seedlings were significantly smaller at 82 μm and 103 μm in the presence of 1.0 mM or 0.3 mM selligueain A, respectively (Table 2 and Fig 4). The inhibitory activity of selligueain A on metaxylem cell size ranged from −51% to −14% from highest to lowest concentration (Table 1), with an IC_50_ value of 0.98 mM (R^2^ = 0.96).

**Table 2:** Relative frequencies (%) of the size classes of sesame (*S*. *indicum*) seedling root metaxylem cells treated with different concentrations of selligueain A.

Size Class (μm)	0 |- 50	50 |- 80	80 |- 110	110 |- 140	140 |- 170	170 |-200	200 |- 230	230 |-260	260 |- 290
Control	0	0	0	25	40	20	7.5	2.5	5
0.01 mM	0	5	22.5	20	27.5	15	7.5	2.5	0
0.03 mM	0	2.5	25	32.5	27.5	10	2.5	0	0
0.1 mM	0	7.5	20	30	22.5	17.5	2.5	0	0
0.3 mM	0	12.5	52.5	30	5	0	0	0	0
1 mM	7.5	37.5	45	10	0	0	0	0	0

Chlorophyll content is related to light capture efficiency [48], and as chlorophyll a has several physiological functions in the plant, this biochemical parameter can reflect growth disruptions and toxic stress [49]. Although early growth inhibition was observed, selligueain A showed no inhibitory activity against the chlorophyll content of the sesame seedlings tested (Table 3).

**Table 3:** Chlorophyll a (C_a_), chlorophyll b (C_b_), and total chlorophyll (C_a + b_) contents^a^ of photosynthetic cotyledons of sesame (*S*. *indicum*) seedlings treated with different concentrations of selligueain A.

Selligueain A (mM)	C_a_	C_b_	C_a+b_
Control	1.05 ± 0.11 NS	0.18 ± 0.02 NS	1.25 ± 0.13 NS
0.01	1.00 ± 0.03 NS	0.19 ± 0.02 NS	1.19 ± 0.03 NS
0.03	1.07 ± 0.03 NS	0.18 ± 0.02 NS	1.26 ± 0.04 NS
0.1	0.95 ± 0.08 NS	0.19 ± 0.01 NS	1.13 ± 0.09 NS
0.3	1.11 ± 0.16 NS	0.18 ± 0.01 NS	1.30 ± 0.18 NS
1	1.06 ± 0.08	0.20 ± 0.03	1.24 ± 0.09
Statistics	F (p)^b^	X^2^ (p)^c^	F (p)
	1.18 (0.36)	3.30 (0.65)	0.90 (0.50)

NS = Difference non-significant from control

^a^ Chlorophyll content expressed as chlorophyll milligrams per fresh tissue mass (mg.g^−1^ ± standard deviation)

^b^ F = ANOVA F-statistic; p = probability

^c^ X^2^ = Kruskal–Wallis H-statistic; p = probability.

Selligueain A showed phytotoxic activity against the early growth of sesame, affecting stem and root size in particular. At a cellular level, root growth inhibition is caused by a reduction in root cell elongation, at least for the two higher concentrations tested (1 mM and 0.3 mM). The inhibition observed at lower concentrations may be due to cell division disruption, although this mechanism was not assessed in this study [46]. It is possible that outcomes from laboratory studies may not reproduce under field conditions because environmental factors may interfere with Selligueain A activity. Nevertheless, this is the first evidence of the potential for phytotoxicity and allelopathy from a compound isolated and identified from *P*. *arachnoideum* or any member of the *Pteridium* species complex, supporting the role of allelopathy in this plant’s dominance.

### Soil Analysis

Despite the evidence from our laboratory experiments suggesting phytotoxic activity and allelopathic potential of selligueain A, the compound can only be considered as an allelochemical if it is present in the environment and available in the soil under natural conditions during the development of the target species [22,50].

**Fig 5:**
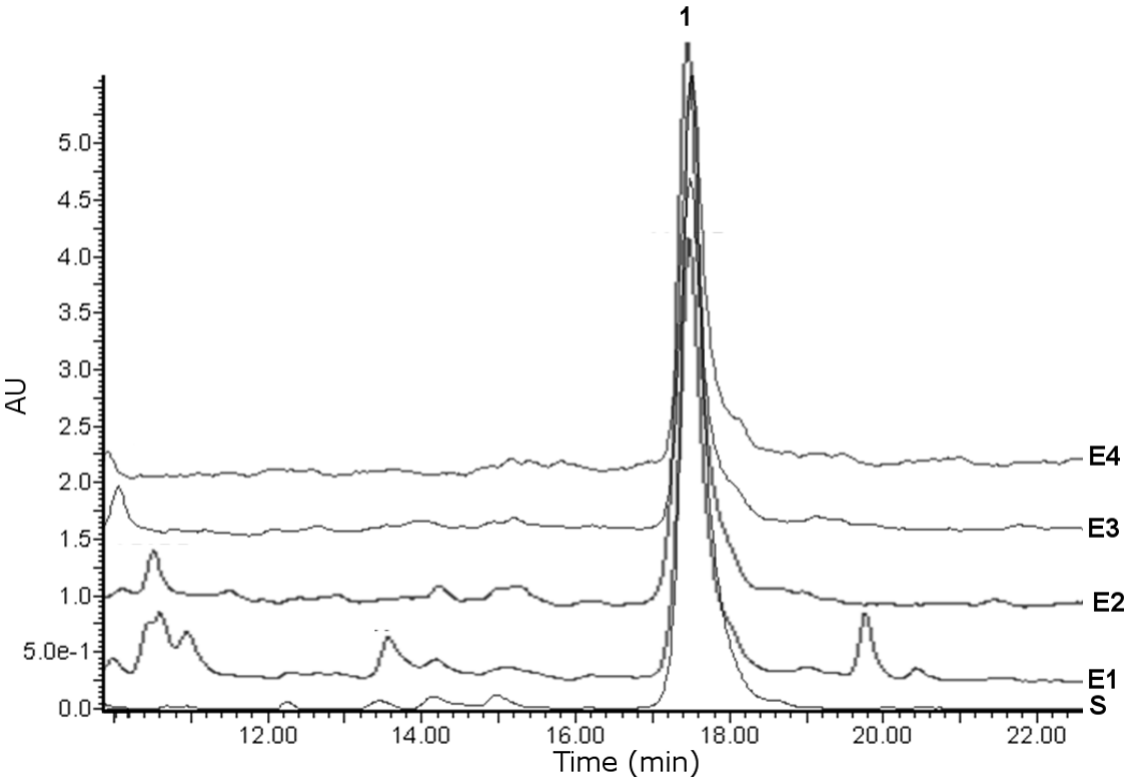
HPLC-PDA chromatograms of the extracts of soil collected from under a *P*. *arachnoideum* patch. S: selligueain A standard; E1: ESQ-1 soil sample extract; E2: ESQ-2 soil sample extract; E3: ESQ-3 soil sample extract; E4: ESQ-4 soil sample extract; 1: selligueain A peak.

HPLC-PDA analysis of the isolated selligueain A used as a standard showed this compound as a chromatographic peak at 17.50 s (Fig 5), with UV absorption at 272 nm (H_2_O/ACN 5:2 v/v). For the soil sample extracts, selligueain A was identified as one of the major compounds in the solution of four independent soil samples (Fig 5).

Plant-produced compounds are released in the environment by several mechanisms. Of these, lixiviation from green fronds and litter and litter decomposition are the two most likely to be involved in the release of selligueain A from *P*. *arachnoideum* [50].

Allelopathic activity has been attributed to phenolic acids associated with the root system of *P*. *aquilinum* and to green frond leachates of *P*. *esculentum* [51,52]. The results reported herein corroborate the hypothesis of allelopathy in bracken fern with the identification of a phytotoxic condensed tannin, a polyphenolic compound produced by the green fronds of *P*. *arachnoideum* and present as a major secondary metabolite in frond litter. This compound may then be leached or liberated by litter decomposition into the soil [16] to be found associated with the plant’s root system but not present within the plant’s rhizome tissue.

Efforts made to control *Pteridium* invasions, including control and restoration experiments conducted in the UK and Norway, have shown that in the long-term, mechanical cutting twice a year and litter removal were the best approaches for controlling *P*. *aquilinum* and promoting higher species richness [4,8,53]. Bracken litter depth and cover influenced seed bank composition in acid grasslands and heathlands in the UK, acting as a barrier that interfered with seed germination or impeded seeds from the seed rain from reaching the soil [7]. The effects of neotropical bracken on the seed bank composition in the Atlantic Forest in Brazil [11] may also be associated with litter cover. However, in addition to the mechanical effect, the new evidence presented herein suggests that the removal of litter as a source of allelochemicals may also be associated with the observed improvement in species richness [4]. Accordingly, more research is needed to establish whether the phenotypic plasticity within the *Pteridium* genus [1,2] influences their chemical composition, with special attention given to condensed tannins present in the green fronds and frond litter. This information could lead to the development of more efficient control protocols for *Pteridium* for the purposes of ecological restoration.

## Conclusions

This study expands on our knowledge of the phytochemical constituents of *Pteridium arachnoideum*, reporting for the first time that proanthocyanidin selligueain A is present in a member of the Dennstaedtiaceae family. Likewise, this is the first time an isolated and identified putative allelochemical compound produced by a member of the *Pteridium* species complex has been described. Selligueain A is produced by *P*. *arachnoideum* in its green fronds, remains unaltered during the senescence process, and is the major secondary compound in the litter. This compound showed phytotoxic activity against the early development of sesame seedlings, inhibiting root and stem growth and root metaxylem cell size. Selligueain A can be considered as an allelochemical because it is present in the soil under *P*. *arachnoideum* as one of the major compounds in the soil solution. This evidence of selligueain A as a putative allelochemical produced by *P*. *arachnoideum* indicates that allelopathy is involved in this plant’s dominance in the areas where it occurs.

## Supporting Information

S1 File NMR (MeOD_4_) spectrum of selligueain A from green fronds of *P*. *arachnoideum*. (PDF)

S2 File ^1^H NMR (MeOD_4_) of the L-3 fraction of the EtOAc extract of *P*. *arachnoideum* litter. (PDF)

S3 File ^1^H NMR (MeOD_4_) of selligueain A from the litter of *P*. *arachnoideum*. (PDF)
