# Supplementary figures and images for: Allelopathy of Bracken Fern (Pteridium arachnoideum): New Evidence from Green Fronds, Litter, and Soil
Source: PLoS One. 2016 Aug 23;11(8):e0161670. doi: 10.1371/journal.pone.0161670 (PMC4995010; doi:10.1371/journal.pone.0161670)

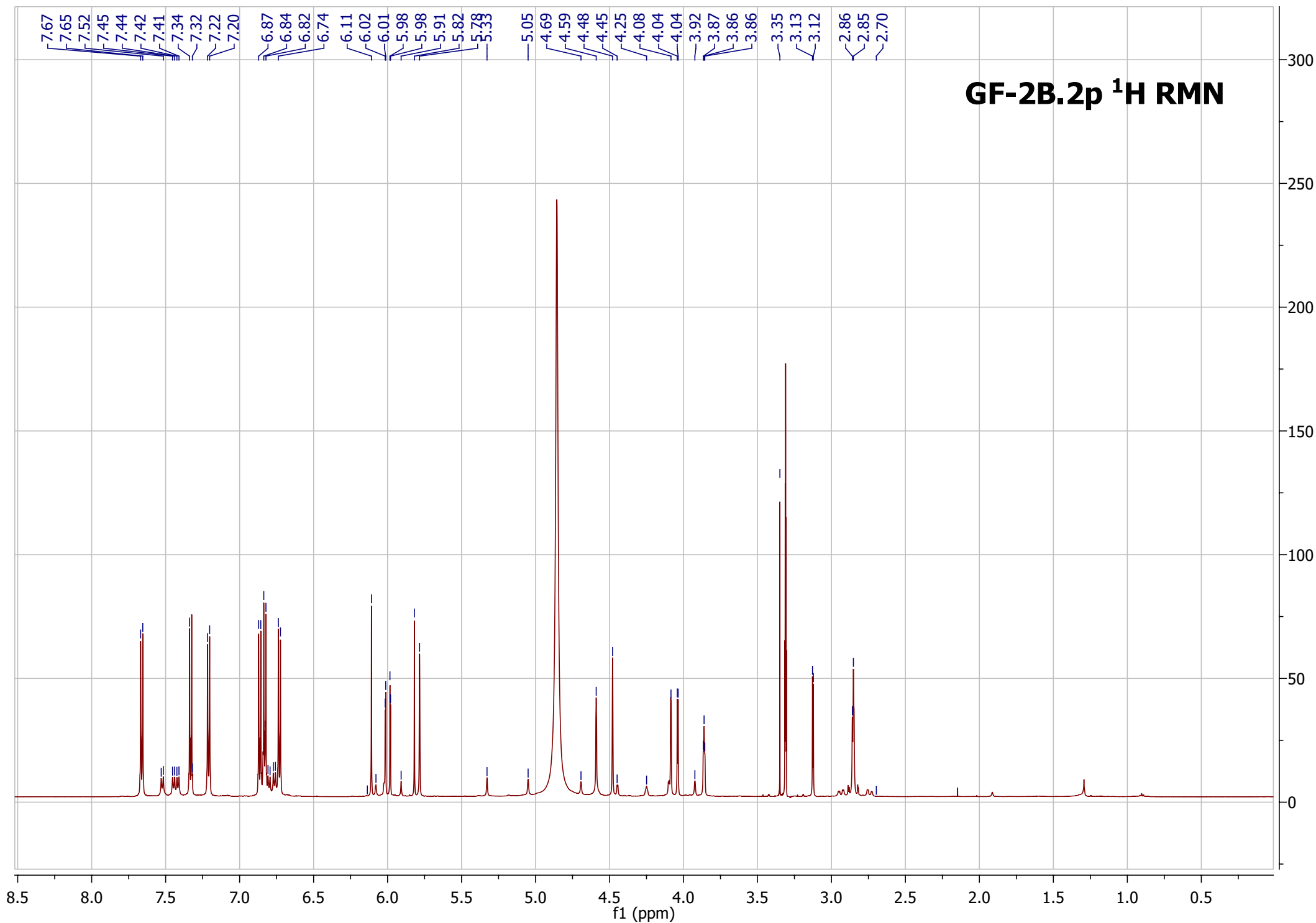

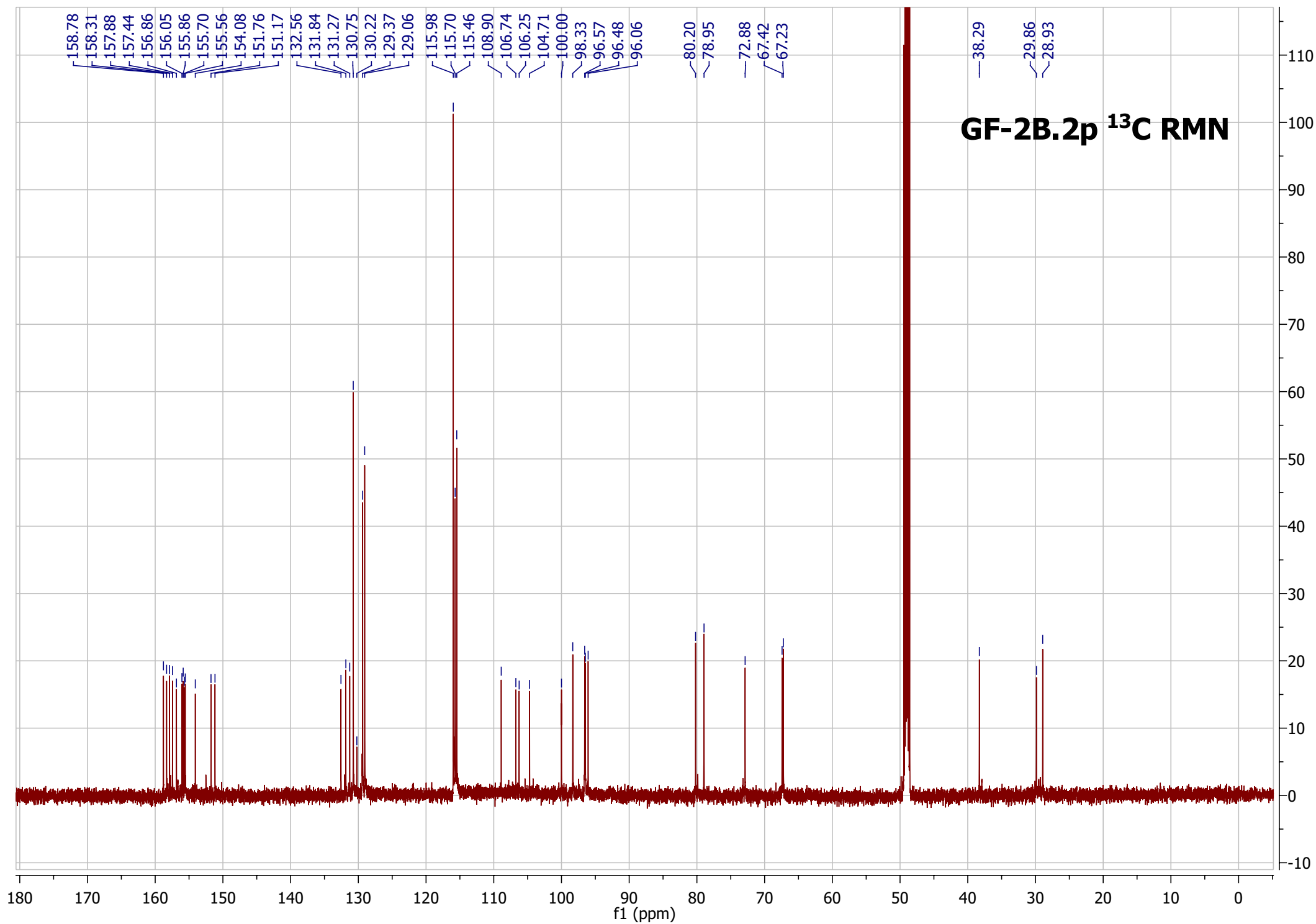

GF-2B.2p COSY  $^1\text{H}$ - $^1\text{H}$  RMN

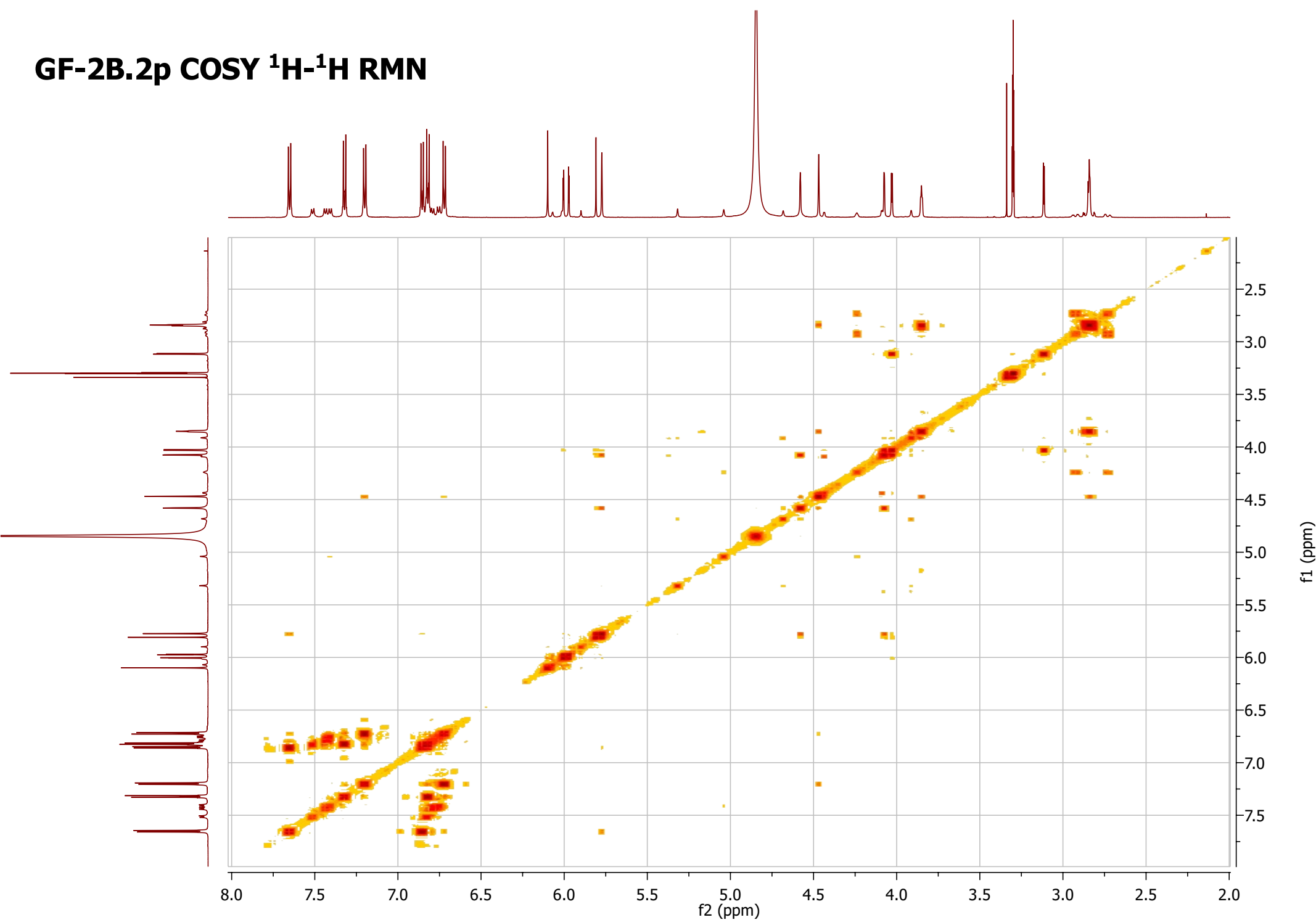

# GF-2B.2p HSQC RMN

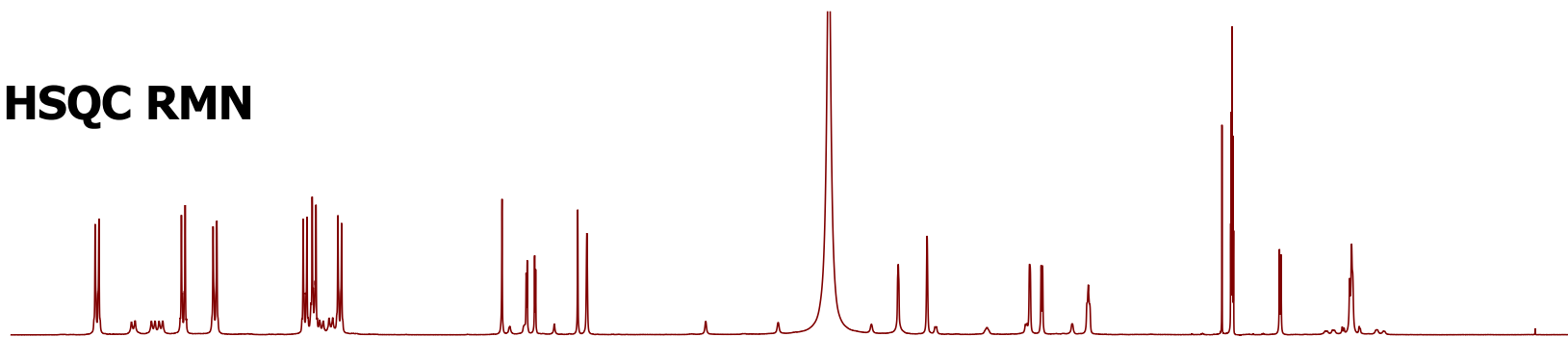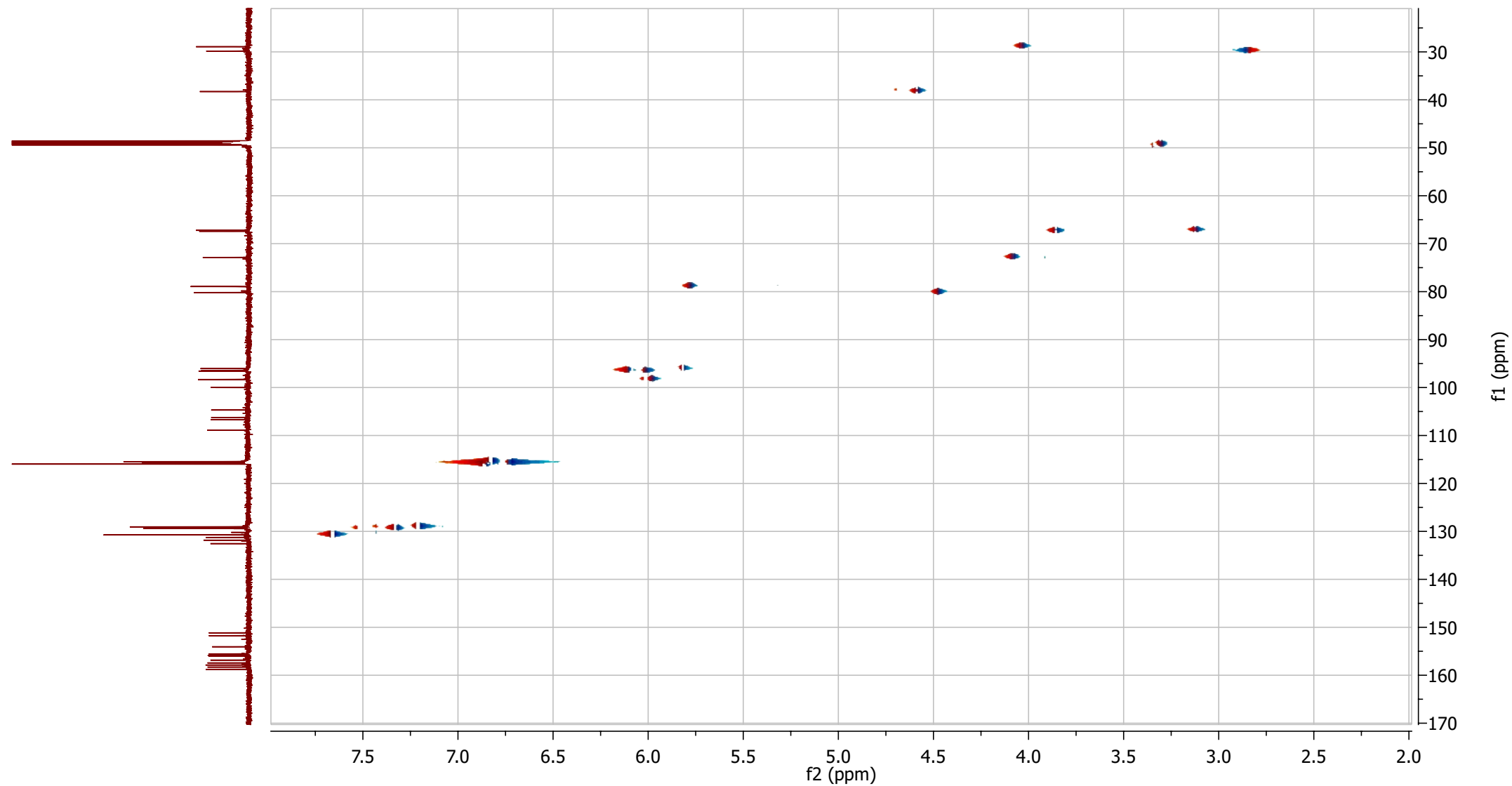

# GF-2B.2p HMBC

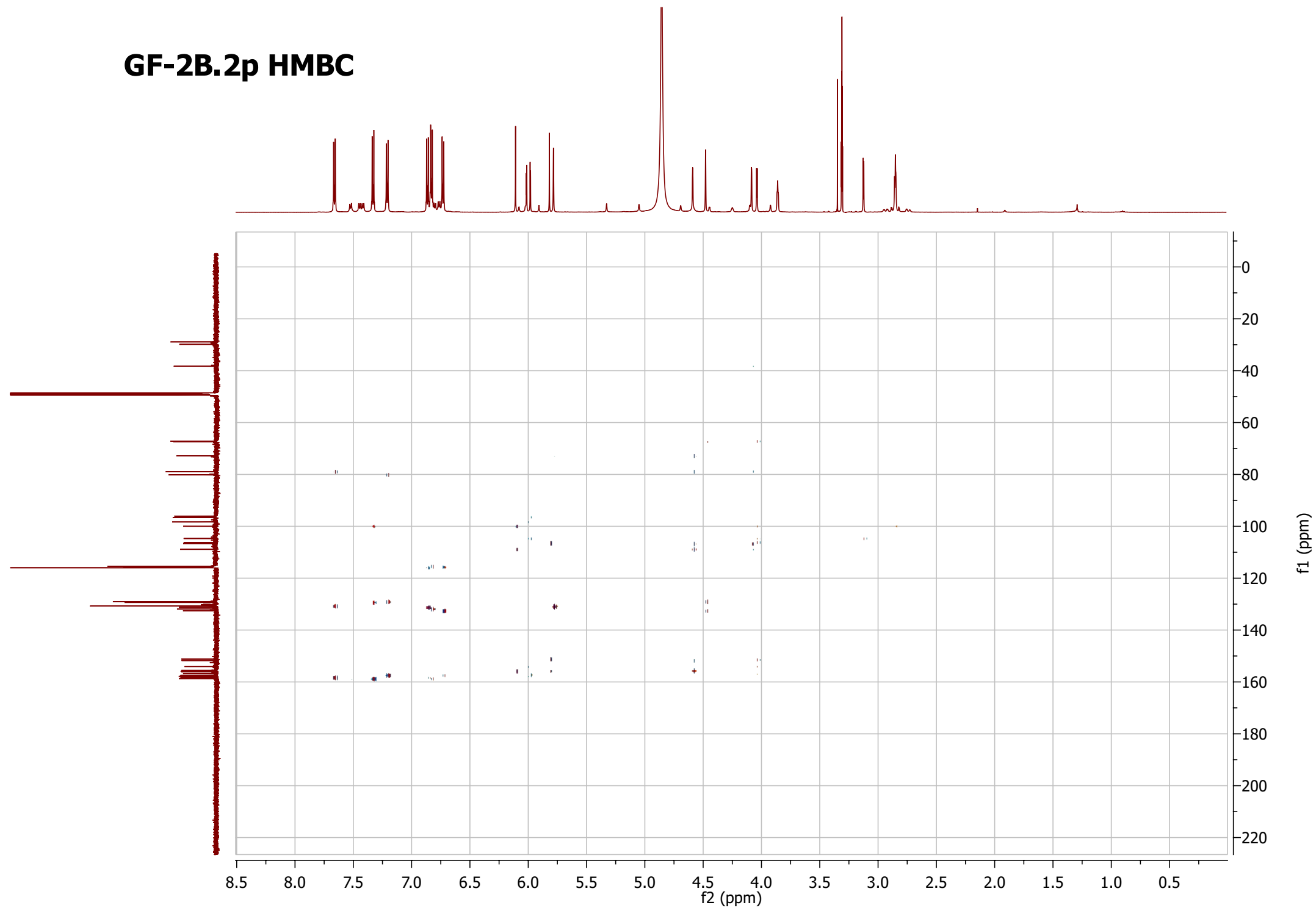

Supplement: S1 File — (PDF) [file pone.0161670.s001.pdf]

# L3 $^1\text{H}$ RMN

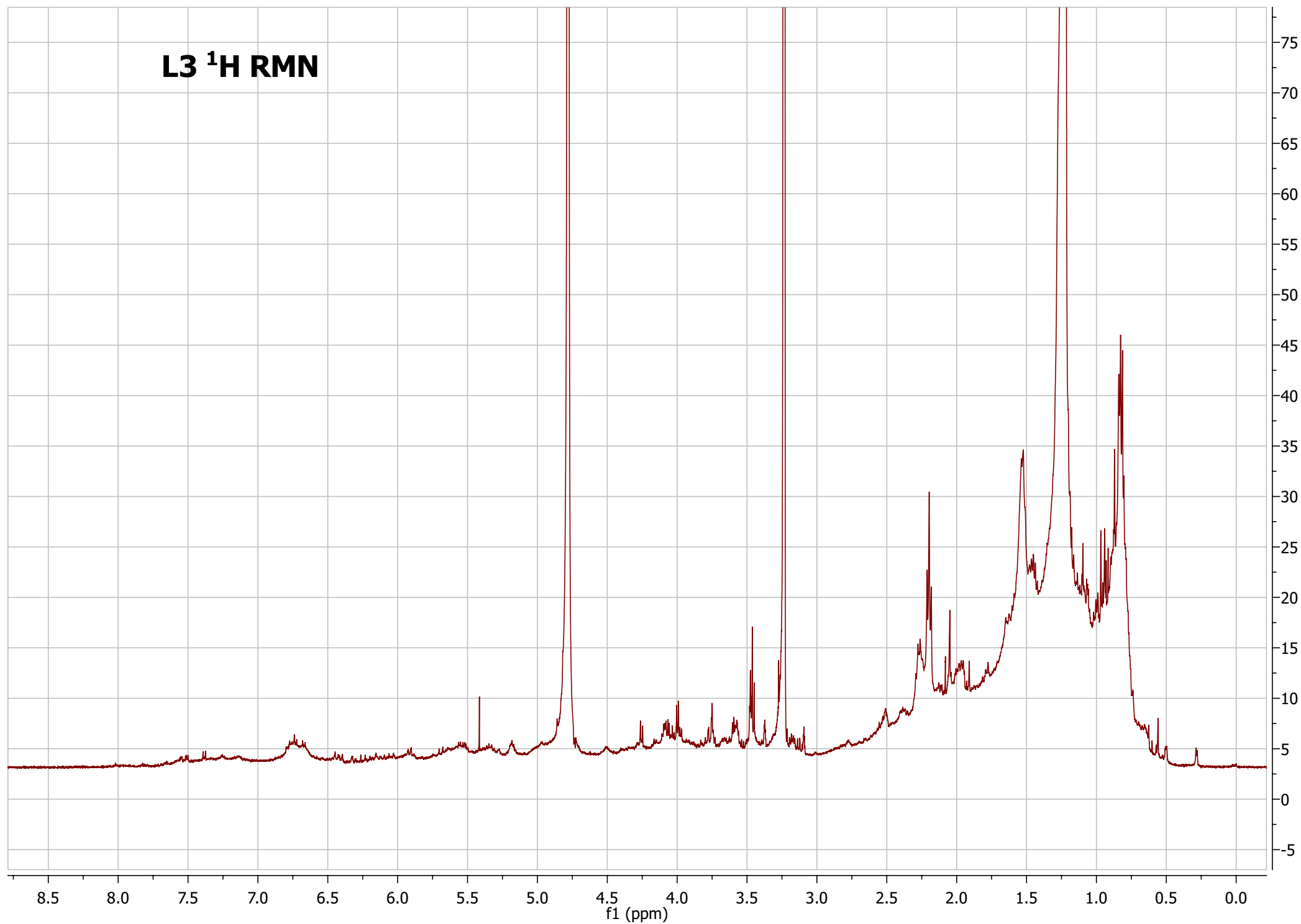

Supplement: S2 File — (PDF) [file pone.0161670.s002.pdf]

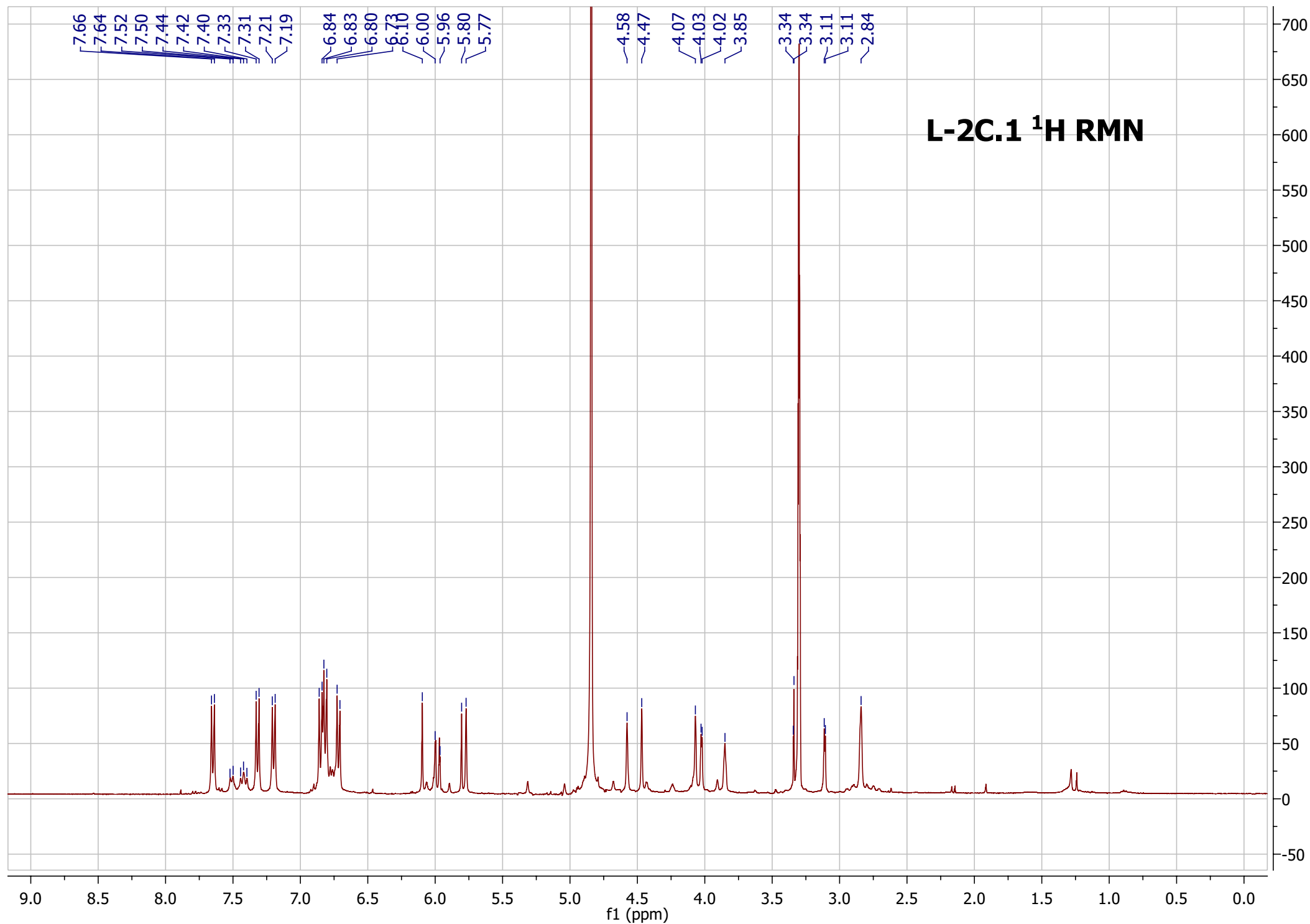

Supplement: S3 File — (PDF) [file pone.0161670.s003.pdf]
